# Dosimetric comparison of preoperative single‐fraction partial breast radiotherapy techniques: 3D CRT, noncoplanar IMRT, coplanar IMRT, and VMAT

**DOI:** 10.1120/jacmp.v16i1.5126

**Published:** 2015-01-08

**Authors:** Sua Yoo, Rachel Blitzblau, Fang‐Fang Yin, Janet K. Horton

**Affiliations:** ^1^ Department of Radiation Oncology Duke University Medical Center Durham NC USA

**Keywords:** single‐fraction radiotherapy, partial breast radiotherapy, treatment planning

## Abstract

The purpose of this study was to compare dosimetric parameters of treatment plans among four techniques for preoperative single‐fraction partial breast radiotherapy in order to select an optimal treatment technique. The techniques evaluated were noncoplanar 3D conformal radiation therapy (3D CRT), noncoplanar intensity‐modulated radiation therapy (${\text{IMRT}}_{\text{NC}}$), coplanar IMRT (${\text{IMRT}}_{\text{CO}}$), and volumetric‐modulated arc therapy (VMAT). The planning CT scans of 16 patients in the prone position were used in this study, with the single‐fraction prescription doses of 15 Gy for the first eight patients and 18 Gy for the remaining eight patients. Six (6) MV photon beams were designed to avoid the heart and contralateral breast. Optimization for IMRT and VMAT was performed to reduce the dose to the skin and normal breast. All plans were normalized such that 100% of the prescribed dose covered greater than 95% of the clinical target volume (CTV) consisting of gross tumor volume (GTV) plus 1.5 cm margin. Mean homogeneity index (HI) was the lowest (1.05±0.02) for 3D CRT and the highest (1.11±0.04) for VMAT. Mean conformity index (CI) was the lowest (1.42±0.32) for ${\text{IMRT}}_{\text{NC}}$ and the highest (1.60±0.32) for VMAT. Mean of the maximum point dose to skin was the lowest (73.7±11.5%) for ${\text{IMRT}}_{\text{NC}}$ and the highest (86.5±6.68%) for 3D CRT. ${\text{IMRT}}_{\text{CO}}$ showed very similar HI, CI, and maximum skin dose to ${\text{IMRT}}_{\text{NC}}$ (differences<1%). The estimated mean treatment delivery time, excluding the time spent for patient positioning and imaging, was 7.0±1.0,8.3±1.1,9.7±1.0, and 11.0±1.5min for $\text{VMAT},{\text{IMRT}}_{\text{CO}},{\text{IMRT}}_{\text{NC}}$ and 3D CRT, respectively. In comparison of all four techniques for preoperative single‐fraction partial breast radiotherapy, we can conclude that noncoplanar or coplanar IMRT were optimal in this study as IMRT plans provided homogeneous and conformal target coverage, skin sparing, and relatively short treatment delivery time.

PACS numbers: 81.40.Wx, 87.55.D‐

## I. INTRODUCTION

Postoperative accelerated partial breast irradiation (APBI) using an external beam technique has been commonly used to treat low‐risk early breast cancer patients with a smaller volume and larger fractional dose than standard whole breast irradiation (WBI).^1^, ^2^ However, outcome data raise concern that soft tissue fibrosis and cosmetic outcomes may be suboptimal with the external beam technique, possibly related to the large volume of tissue treated to high doses in the postoperative setting.^3^, ^4^, ^5^ In contrast, a single large dose using intraoperative radiation therapy (IORT) technique has shown encouraging results in toxicity and cosmesis outcomes.^6^, ^7^ While IORT application is limited due to the costly equipment and specialized procedures, a radiosurgery technique using external beam is easily performed by most radiation facilities. Feasibility of single‐dose partial breast irradiation using a radiosurgery technique has been investigated and shown promising results.^8^, ^9^, ^10^, ^11^ Treatment of the intact tumor preoperatively allows a significant reduction in high‐dose treatment volume and direct access to more accurately defined high‐risk target area than postoperative seroma.^11^, ^12^ It also provides an opportunity to assess the response of breast tumors to radiation. For such reasons, a phase I clinical study to evaluate preoperative single‐fraction partial breast radiotherapy was proposed for early‐stage breast cancer patients and approved by the institutional review board at Duke University.

Given the relative novelty of this approach, the optimal treatment delivery technique was unknown. Although 3D CRT has been traditionally used for postoperative APBI,^1^, ^13^, ^14^ significantly reduced dose to normal surrounding tissue and critical structures with more conformal target coverage have been reported in studies using IMRT or VMAT techniques.^15^, ^16^, ^17^, ^18^, ^19^, ^20^ However, all prior IMRT or VMAT studies for breast patients did not include constraints for the skin, which, we were concerned, could be a major limitation for single‐fraction breast radiotherapy.

The purpose of this study was to compare dosimetric parameters of treatment plans in order to select an optimal treatment technique for preoperative single‐fraction partial breast radiotherapy. The techniques we evaluated in this study were noncoplanar 3D conformal radiation therapy (3D CRT), noncoplanar intensity‐modulated radiation therapy (${\text{IMRT}}_{\text{NC}}$), coplanar IMRT (${\text{IMRT}}_{\text{CO}}$), and volumetric‐modulated arc therapy (VMAT).

## II. MATERIALS AND METHODS

### A. Simulation

This study included 16 patients who enrolled on a preoperative, dose escalation, single‐fraction partial breast radiotherapy clinical protocol at Duke. Prior to CT scan, all patients had one titanium biopsy marker implanted to identify the tumor. Patients underwent CT (GE LightSpeed RT; GE Medical System, Milwakee, WI) scanning with 2.5 mm slice thickness in the prone position on a CDR prone breast board (CDR Systems Inc, Calgary, AB, Canada), which was placed on top of the CT table and treatment couch. The head was turned to the ipsilateral side and both arms were raised up to hold the handles above the head. The ipsilateral breast (ILB) was allowed to fall naturally through the opening of the prone breast board while the contralateral breast^(21)^ was pulled away with the support of the bridge plate on the board.^(22)^ CT origin was marked on the patient's skin and the board. Patients also underwent MRI in the prone position (1.5 Tesla GE Signa scanner; GE Medical System) using the open breast array MRI coil (GE Medical System). T1 weighted and dynamic contrast‐enhanced MR images were acquired to delineate the tumor.

### B. Structure segmentations

CT and MR images were imported into the Eclipse treatment planning system (TPS) (Varian Medical Systems, Inc., Palo Alto, CA) and registered to align the biopsy marker and soft tissue around the tumor area using manual rigid‐body registration. All structures were contoured on the CT. Gross tumor volume (GTV) was identified as the area of enhancement on contrast‐enhanced MRI with the assistance of a radiologist specializing in breast imaging.^(23)^ Additional images (mammogram and ultrasound) were also viewed for accuracy and the biopsy marker was confirmed to be in the region of the tumor. A 1.5 cm margin and an additional 0.3 cm margin from the GTV were added to define the clinical target volume (CTV) and the planning target volume (PTV).^9^, ^10^, ^11^ Both CTV and PTV were modified to keep a minimum 5 mm distance from the skin surface to ensure skin sparing, following RTOG protocol 0413^(24)^ and other single‐fraction breast radiotherapy studies.^9^, ^25^ It is important to note that the CTV margins for this application were more comprehensive than other single‐fraction conformal applications.^8^, ^9^, ^25^ Furthermore, a single‐fraction delivery technique using on‐board imaging and target localization based on biopsy marker contributed to minimize patient motion. Therefore, a priority was given to skin sparing over additional margin when generating the PTV. A 3 mm layer along the ILB skin surface was defined as skin, following a previous study of breast single‐fraction radiotherapy.^(9)^ Heart, lungs, ribs, and bilateral normal breasts were segmented on the CT.

### C. Treatment planning and optimization

All plans used the anisotropic analytical algorithm (AAA version 10.0.28) for dose calculation with 2.5 mm calculation grid and heterogeneity correction. We intended to set the beam isocenter at the geometrical center of GTV. However, for some patients, the isocenter was set a few centimeters (3 to 8 cm) medial to this point in order to avoid collision between the gantry and the patient body/treatment couch. All plans had gantry, collimator, and couch angles set to avoid CB and heart based on beams eye view (BEV) in TPS. No beams entered from the back or side of a patient through a lung. All beams entered directly through the ipsilateral breast to reach to the target effectively. In general, the gantry angles were set to be spread as much as possible (15° to 40°) among beams within the limited range. If a target was located at the periphery, more beams were arranged to enter from the target side, and one to two beams were arranged to enter from the other sides. TrueBeam (Varian Medical Systems) 6 MV photon beams with 600 MU/min dose rate were used for treatment plan dose calculation. All plans were normalized such that 100% of the prescribed dose covered greater than 95% of the CTV (i.e., $V_{100\%}>95\%$). The single‐fraction prescription dose was 15 Gy for the first eight patients and 18 Gy for the remaining patients (first two dose cohorts on the clinical trial).

#### C.1 3D CRT

Noncoplanar 3D CRT included four to five beams following typical APBI 3D CRT planning,^1^, ^14^ among which one to three beams included wedges. The couch rotation was applied to one to three beams with a limited angle up to ±20° to avoid collision. Multileaf collimators (MLC) were set to have a 2 mm margin around the PTV or tighter if critical organs such as skin, CB, or heart were abutting to the PTV based on BEV. Beam weights and wedge angles were adjusted to provide homogeneous dose distribution and conformal dose to the CTV and PTV.

#### C.2 Noncoplanar IMRT (${\text{IMRT}}_{\text{NC}}$)

Noncoplanar IMRT used dynamic MLC with the same beams utilized for 3D CRT. Optimization was performed to minimize dose to skin and ILB. Optimization constraints and priorities from Table 1 were initially applied, and they were adjusted as optimization progressed to improve target coverage and to reduce skin dose. Dose‐volume constraints for heart, CB, and ipsilateral lung (ILL) were set with relatively low priority. No constraint was set for ribs. Optimization process was stopped after about 50‐70 iterations, as the optimization process did not show improved dose‐volume histograms (DVH).

**Table 1:** Initial dose‐volume constraints and priorities for IMRT and VMAT optimization

*Structure*	*Dose‐volume Constraints*	*Relative Priority*
PTV	$D_{100}\geq 95\%$;$D_{\text{max}}\leq 105\%$	80–120
CTV	$D_{100}\geq 98\%$;$D_{\text{max}}\leq 102\%$	120–130
	$D_{\text{max}}\leq 70\%$;	150
Skin	$V_{50\%}\leq^{2\%}$	80
ILB	$V_{100\%}\geq 2\%–7\%$; $V_{50\%}\geq 10\%–15\%$; $V_{20\%}\leq 20\%‐30\%$;	50
CB	$D_{\text{max}}\leq 1\%$;	30
Heart	$D_{\text{max}}\leq 1\%$;	30
ILL	$D_{\text{max}}\geq 20\%–30\%$; $V_{10\%}\leq 2\%–7\%$	30
Normal tissue	Doses between 2 mm and 2 cm from PTV boundary to fall off from 100% to 50% of prescription	80

#### C.3 Coplanar IMRT (${\text{IMRT}}_{\text{CO}}$)

Coplanar IMRT included seven beams with couch angle at zero, also using dynamic MLC. The same optimization constraints and process from ${\text{IMRT}}_{\text{NC}}$ planning were used.

#### CA VMAT

VMAT is a volumetric arc therapy, which utilizes IMRT technique with different MUs delivered at varying dose rate while MLC leafs continuously moving as the gantry is simultaneously rotating at varying speed.^(26)^ RapidArc (Varian Medical Systems) in Eclipse was used for VMAT planning in this study. Two duplicate sets of partial arcs were included — each set divided into two or three partial arcs to avoid any angle passing through heart and CB. One set rotated counterclockwise with collimator rotation of 30° and the other set rotated clockwise with collimator rotation of 330°. The same optimization constraints and process from ${\text{IMRT}}_{\text{NC}}$ and ${\text{IMRT}}_{\text{CO}}$ planning were used.

### D. Data analysis

For CTV and $\text{PTV},V_{95\%}$, dose homogeneity index (HI), and conformity index (CI) were compared. The ratio of a maximum point dose ($D_{\text{max}}$) within the target and the prescribed dose (PD), $D_{\text{max}}/\text{PD}$, defined the HI.^(27)^ The ratio of prescribed isodose volume (PIV) to the target volume,^(7)^ PIV/TV, defined the CI.^(27)^ The skin $D_{\text{max}}$ and dose to $10\;{\text{cm}}^{3}(D_{10}\;{\text{cm}}^{3})$ of skin, as well as $V_{20\%},V_{50\%},V_{100\%}$, and $D_{1\%}$ of ILB, were compared. For heart, CB, and $\text{ILL},D_{10}\;{\text{cm}}^{3}$ was compared. The dose falloff was calculated as the ratio of $V_{50\%}$ of ILB to $V_{100\%}$ of $\text{ILB},V_{50\%}$/$V_{100\%}$.

Treatment delivery time was estimated by adding beam‐on time and time in between beams. The beam‐on time was calculated as the total MUs divided by the dose rate. Even though VMAT utilized a varying dose rate, the maximum dose rate was used for calculation in this study because each arc beam was irradiating the small target with small arc angle and large MUs. To include beam preparation and therapists’ time‐out procedure, 20 sec was added for each beam. For a beam with a wedge or couch rotation, 1 min was added to include the time for a therapist to go in and out of the treatment room. For a beam with a wedge and couch rotation, 1 min and 20 sec was added to apply both. Time spent for patient positioning and image guidance was not included in estimating the treatment delivery time.

Comparison of the different techniques was statistically analyzed using the two‐tailed paired *t*‐test (p value). Data were considered significant if *p* was < 0.05.

## III. RESULTS

The average volumes of GTV, CTV, and PTV were $1.3\pm 0.8\;{\text{cm}}^{3}$ (range 0.2–3.5 cm^3)^, $46.0\pm 9.3\;{\text{cm}}^{3}$ (range 33.9–65.9 cm^3)^, and $68.5\pm 12.9\;{\text{cm}}^{3}$ (range 52.3–96.5 cm^3)^, respectively. The average volume of ILB was $1905.2\pm 829.6\;{\text{cm}}^{3}$ (range 775.9–3688.8 cm^3)^ . The average volume ratio of PTV to ILB was 4.1%±1.5% (range 1.5%–7.1%).

For target coverage, all plans had $V_{95\%}$ of CTV higher than 99.5% and $V_{95\%}$ of PTV higher than 95%. 3D CRT had the highest $V_{95\%}$ of CTV and PTV. VMAT had higher CIs and HIs of CTV and PTV than other three techniques. For skin sparing, ${\text{IMRT}}_{\text{NC}}$ had the lowest $D_{10}\;{\text{cm}}^{3},D_{1}\;{\text{cm}}^{3}$, and $D_{\text{max}}$. Skin $D_{1}\;{\text{cm}}^{3}$ and $D_{\text{max}}$ was the highest for 3D CRT, and skin $D_{10}\;{\text{cm}}^{3}$ was the highest for VMAT. Regarding ILB, VMAT had the lowest $V_{20\%}$ and $V_{50\%}$, but the highest $V_{100\%}$ and $D_{1\%}$. ${\text{IMRT}}_{\text{NC}}$ and ${\text{IMRT}}_{\text{CO}}$ showed similar target conformity, dose homogeneity, and normal tissue sparing. 3D CRT plans yielded higher doses to skin and ILB, but had better target coverage and dose homogeneity than other plans. Heart, CB, and ILL received very small doses from all plans in this study. VMAT had the smallest $D_{10}\;{\text{cm}}^{3}$ for heart, CB, and ILL compared to the other techniques. The dose falloff was sharper for VMAT than other techniques. The estimated treatment delivery time was about 4 min faster for VMAT than 3D CRT. ${\text{IMRT}}_{\text{CO}}$ and ${\text{IMRT}}_{\text{NC}}$ were estimated to require 1.3 min and 2.7 min more than VMAT, respectively.

**Figure 1:**
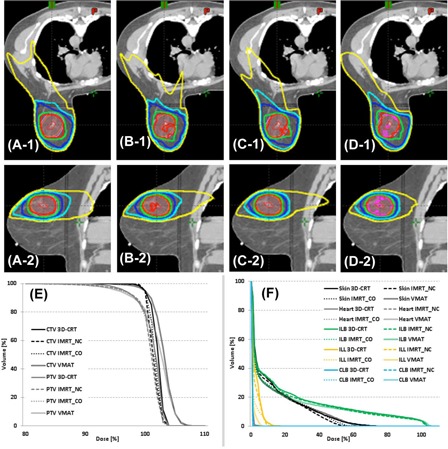
Isodose distributions of 3D CRT (A‐1 & A‐2), ${\text{IMRT}}_{\text{NC}}$ (B‐1 & B‐2), ${\text{IMRT}}_{\text{CO}}$ (C‐1 & C‐2), and VMAT (D‐1 & D‐2) in axial view (top row) and sagittal view (bottom row), as well as dose‐volume histograms of CTV and PTV (E) and normal tissues (F). Magenta=105%,red=100%,green=90%,blue=50%,cyan=30%, and yellow=10% isodose lines; GTV is contoured in red, CTV in orange, and PTV in pink.

In the isodose comparison as shown in Fig. 1 for one patient, it is notable that the 105% isodose line (magenta) appears only in VMAT, as anticipated, based on the HI comparison from Table 2. VMAT also shows a smaller 10% isodose line (yellow) than other plans, as anticipated, given the findings from ILB's dose comparison (e.g., $V_{20\%}$) presented in Table 2. DVHs for this patient are also compared in Fig. 1 (E) and (F).

**Table 2:** Mean dosimetric parameters ± standard deviation from 3D CRT, ${\text{IMRT}}_{\text{NC}},{\text{IMRT}}_{\text{CO}}$, and VMAT

	*3D CRT*		${\text{IMRT}}_{\text{NC}}$	${\text{IMRT}}_{\text{CO}}$	*VMAT*
CTV	$V_{95\%}$	99.8±0.4	99.5±0.6 ^*^	99.5±0.6 ^*^	99.5±0.6 ^*^
	HI	1.05±0.02	1.07±0.04 ^*^	1.07±0.03 ^*^	1.11±0.04 ^*†‡^
	CI	1.56±0.27	1.42±0.32	1.44±0.30	1.60±0.32
PTV	$V_{95\%}$	98.5±1.4	96.3±2.0 ^*^	96.5±1.5 ^*^	96.4±1.8 ^*^
	HI	1.05±0.02	1.08±0.05 ^*^	1.07±0.04 ^*^	1.12±0.02 ^*†‡^
	CI	1.04±0.17	0.95±0.20	0.96±0.21	1.07±0.22 ^†‡^
Skin	$D_{10}\;{\text{cm}}^{3}\;(\%)$	45.9±7.1	41.9±5.9 ^*^	43.8±6.5	46.3±7.42 ^†‡^
	$D_{1}\;{\text{cm}}^{3}\;(\%)$	72.9±9.6	59.2±10.3 ^*^	61.1±11.9 ^*^	64.2±11.7 ^*†^
	$D_{\text{max}}\;(\%)$	86.5±6.7	73.7±11.5 ^*^	74.3±12.1 ^*^	76.3±12.5 ^*^
ILB	$V_{20\%}\;(\%)$	28.5±8.5	27.0±7.8_*_	26.4±8.3_*_	23.5±7.5 ^*†‡^
	$V_{50\%}\;(\%)$	15.3±5.3	14.3±3.9	14.8±5.5	14.1±5.6 ^*†^
	$V_{100\%}\;(\%)$	4.3±1.6	3.8±1.3 ^*^	3.9±1.4	4.4±1.9 ^†‡^
	$D_{1\%}\;(\%)$	103.7±1.8	103.9±2.5 ^*^	104.0±2.3 ^*^	105.6±2.8 ^*^
Heart	$D_{10}\;{\text{cm}}^{3}\;(\%)$	2.5±1.7	1.8±1.6 ^*^	1.6±1.4 ^*^	1.3±1.1 ^*†‡^
CB	$D_{10}\;{\text{cm}}^{3}\;(\%)$	1.0±0.5	0.7±0.3_*_	0.8±0.3 ^*†^	0.6±0.2 ^*‡^
ILL	$D_{10}\;{\text{cm}}^{3}\;(\%)$	11.4±8.3	13.2±10.8	11.8±9.8	9.6±9.9 ^†‡^
Ribs	$D_{\text{max}}\;(\%)$	33.6±33.0	34.3±36.6	33.7±35.8	30.4±36.6 ^†‡^
Dose fall off	$V_{50\%}$/$V_{100\%}$ of ILB	3.7±0.8	3.9±0.8	4.0±1.1	3.3±0.8 ^*†‡^
Delivery time (min)		11.0±1.5	9.7±1.0 ^*^	8.3±1.1 ^*†^	7.0±1.0 ^*†‡^

* pvalue<0.05 compared to 3D CRT where p= the two‐tailed paired *t*‐test.

† pvalue<0.05 compared to ${\text{IMRT}}_{\text{NC}}$.

‡ pvalue<0.05 compared to ${\text{IMRT}}_{\text{CO}}$.

## IV. DISCUSSION

The feasibility of single‐fraction partial breast radiotherapy has been previously demonstrated.^8^, ^9^, ^10^, ^11^ Palta et al.^(11)^ used an IMRT technique and treatment planning MR with a prescription dose of 15 Gy preoperatively, whereas the other three studies^8^, ^9^, ^10^ used 3D CRT with a prescription dose of 21 Gy postoperatively based on CT images. Our study investigated further the novel preoperative approach in an effort to reduce the volume of normal tissue treatment^11^, ^12^ with the goal of producing acceptable acute toxicity and cosmetic outcomes.^3^, ^5^ Indeed, our study included PTV with the average volume and the maximum volume 30% and 60%–70% smaller, respectively, than those from the postoperative single‐fraction radiotherapy studies.^9^, ^10^ In addition, our study showed $V_{50\%}$ and $V_{100\%}$ of ILB about 15% and 5%, respectively, whereas postoperative prone APBI studies^28^, ^29^, ^30^ showed about a $V_{50}$ of approximately 40%–50% and $V_{100}$ of ∼20%–30%. Similarly, postoperative supine APBI studies, which reported acceptable cosmetic outcome, showed a $V_{50}$ of ∼35%–40% and $V_{100}$ of ∼10%–15%.^4^, ^31^ Such substantial improvement in $V_{50\%}$ and $V_{100\%}$ of ILB were attributed to the preoperative approach of this study. The studies performed by Bondiau et al.^32^, ^35^ proposed preoperative breast stereotactic body radiotherapy (SBRT) and postoperative conventional breast radiotherapy. Their studies did not report ILB volumes, but showed similar mean PTV volume. Therefore, we presume a similar benefit to preoperative radiotherapy from their studies, as well.

Dose to the skin could be a significant limiting factor for this single‐fraction approach compared to other APBI planning studies. Timmerman^(33)^ suggested 14.4 Gy to <10cc of skin and 16 Gy to skin $D_{\text{max}}$ as dose constraints for single‐fraction treatment to avoid skin ulceration. Our study showed $D_{10}\;{\text{cm}}^{3}$ of skin getting less than 50% of the prescription dose (7.5–9 Gy), but $D_{\text{max}}$ higher than 16 Gy with all four plans for one patient and with 3D CRT for another patient. Bondiau et al.^(32)^ reported Grade 3 dermatologic dose limiting skin toxicity for one patient with the prescription of 17.5 Gy for a 3‐fraction SBRT. They associated this toxicity not with the dose level, but with the tumor size and the sagging patch of skin. Iaccarino et al.^(9)^ reported no skin toxicity with an average of 15.2 Gy to 3% of the skin volume, and our study had less than 15.2 Gy to 3% of the skin volume for all patients with all plans. Pinnaro et al.^(10)^ stopped their study prematurely due to unsatisfactory cosmesis rate or Grade 2 skin toxicity such as erythema, fibrosis, and necrosis, and noted a mean skin dose of ∼5.4Gy. The mean skin dose in our study was less than 3 Gy for all patients with all plans. Hoppe et al.^(34)^ reported acute Grade 2 or higher skin toxicity at skin $D_{\text{max}}$ of 24–30 Gy in 3 to 4 fractions, which is equivalent to 13–18 Gy in a single fraction for skin using an α/β of 2.3. Our study showed skin $D_{\text{max}}>13\;\text{Gy}$ for 15 patients in 3D CRT, —‐five patients in ${\text{IMRT}}_{\text{NC}}$ and VMAT, and four patients in ${\text{IMRT}}_{\text{CO}}$, but all less than 17 Gy. The mean skin $D_{\text{max}}$ was about 1 Gy greater in the 2nd dose cohort (18 Gy) than the 1st dose cohort (15 Gy). Treatment planning may be more challenging for the 3rd dose cohort — 21 Gy. Though this section evaluated our plans compared to other similar studies reporting skin toxicity, it should be noted that the studies from Hoppe and colleagues and from Timmermen were conducted in different clinical scenarios. Though our data compare favorably with other studies, more conservative constraints may be required for this application. Ultimately, we will need to await outcomes from our recently completed phase I study to make this determination. In addition, it should be noted that there could be some uncertainty in the skin dose analysis due to the differences in skin definition. The Timmerman, Pinnaro and Hoppe studies did not specify how skin was defined in their manuscripts, whereas Iaccarino and colleagues used 3 mm and Bondiau et al. used 5 mm thickness to define the skin. Dose calculation accuracy could be another uncertainty in evaluating skin dose, as it is in the buildup region. However, AAA is known to provide accurate dose calculation for the buildup region.^(35)^

Regarding rib fracture, Wilkinson et al.^(36)^ reported 2% of patients getting rib fractures, with the maximum rib dose ranging from 28 to 33.6 Gy delivered over 4 fractions in breast brachytherapy. It is equivalent to 15.3 to18 Gy in a single fraction for rib using an α/β of 3.^(37)^ Our study showed the maximum rib dose of higher than 15.3, but all less than 16.5 Gy for two patients. Dunlap et al.^(38)^ suggested less than 30 ml to receive 30 Gy in 3 to 5 fraction to avoid rib fracture based on lung SBRT. Again, using an α/β of 3, it is equivalent to 15 to 18 Gy in a single fraction. Our study had no patient receiving more than 15 Gy for 0.1 cm^3^ of rib. We anticipate that rib fractures were not a significant risk for patients enrolled in this study. However, we will have to await clinical outcomes from our phase I study for more definitive answers.

Without long‐term follow‐up, it is hard to predict toxicities associated with normal structures such as heart, lung, and CB. This was incorporated into consideration in the designs of the phase I clinical trial and treatment plans. The prescription dose started at 15 Gy for the 1st cohort and increased to 18 Gy in order to follow acute toxicities, and further increased to 21 Gy for the last cohort. Treatment beams in plans were designed to avoid such normal structures, and optimization constraints were also used to limit the dose. Consequently, only small volumes of normal structures received very low dose.

3D CRT and ${\text{IMRT}}_{\text{NC}}$ required at least four or five beams to achieve acceptable target coverage. Adding more beams (total six beams or more) increased the estimated treatment delivery time without noticeable improvement in dosimetric results. VMAT and ${\text{IMRT}}_{\text{CO}}$ have an advantage over 3D CRT and ${\text{IMRT}}_{\text{NC}}$ in that therapists do not need to go in and out of the room in between beams. ${\text{IMRT}}_{\text{CO}}$ was initially tested with various numbers of beams. ${\text{IMRT}}_{\text{CO}}$ with seven beams provided comparable results to ${\text{IMRT}}_{\text{NC}}$ and estimated treatment delivery time fell between VMAT and ${\text{IMRT}}_{\text{NC}}$. VMAT, with one set of partial arcs, was initially intended for this study, but the target coverage was noticeably inferior (e.g., CI>2). VMAT achieved comparable dosimetric results by utilizing duplicate sets of partial arcs. VMAT with triplicate sets of partial arcs showed insignificant improvement in dosimetric parameters compared to VMAT with duplicate sets, while the estimated treatment delivery time increased by 50%. It should be noted that the time spent for patient setup and imaging prior to treatment delivery could be much longer. However, the time increase from IGRT part should be uniform across techniques. Once images are approved by an attending physician, it is critical to deliver the treatment quickly to achieve accurate delivery without patient motion.

Prior APBI plan comparison studies showed that IMRT spared heart, CB, and lungs more than 3D CRT and VMAT, and VMAT spared better than 3D CRT.^15^, ^18^, ^19^ Nevertheless, we included 3D CRT in this study for comparison with other previous single‐fraction breast radiotherapy studies.^8^, ^9^, ^10^ In this study, heart, CB, and lungs received very small dose in all plans. Yet, VMAT had smaller doses to heart, CB, ribs, and ILL than IMRT or 3D CRT due to the reduced scatter with smaller total MUs. Regarding ILB, Shaitelman et al.^(19)^ noted that VMAT plans reduced dose to ILB at the expense of increased dose elsewhere. Our study showed the same trend, that VMAT had sharp dose falloff resulting in reduced dose to ILB, but target dose homogeneities and skin doses were higher compared to IMRT and 3D CRT.

For the actual treatment of the 16 patients, ${\text{IMRT}}_{\text{NC}}$ or ${\text{IMRT}}_{\text{CO}}$ was used. Contrasting to the dosimetric study reported here, beams were allowed to exit through a small portion of the heart and/or CB for some patients. Consequently, the mean $D_{10}\;{\text{cm}}^{3}$ of heart and CB were 5% and 2% greater in the actual plans than in IMRT plans from this study. Doses to other structures in the actual plans were comparable to IMRT plans from this study.

## V. CONCLUSIONS

The treatment planning technique was not specifically delineated in our phase I preoperative single‐fraction partial breast radiotherapy protocol. Therefore, we compared four different techniques to determine the optimal treatment planning technique. All plans achieved acceptable target coverage. 3D CRT yielded significantly higher skin dose than the other plans. VMAT offered the shortest estimated treatment delivery time and better sparing of normal tissue except skin, but yielded less dose homogeneity within target and conformity in this study. Based on the results of our current study, there was no treatment planning technique that provided better dosimetric parameters for all structures with a shortest treatment delivery time. However, we can conclude that either noncoplanar IMRT or coplanar IMRT are the optimal treatment technique for preoperative single‐fraction partial breast radiotherapy, as the IMRT plans provided homogeneous and conformal target coverage and skin sparing with relatively short treatment delivery time.

## Supporting information

Supplementary Material
