# Supplementary material for: Dosimetric comparison of preoperative single‐fraction partial breast radiotherapy techniques: 3D CRT, noncoplanar IMRT, coplanar IMRT, and VMAT
Source: J Appl Clin Med Phys. 2015 Jan 8;16(1):183–207. doi: 10.1120/jacmp.v16i1.5126 (PMC4484297; doi:10.1120/jacmp.v16i1.5126)
Supplement: Supplementary file 1 — Supplementary Material [file ACM2-16-183-s001.doc]

Dosimetric comparison of preoperative single fraction partial breast radiotherapy techniques: 3D CRT, non-coplanar IMRT, coplanar IMRT, and VMAT

Authors have no conflict of interest.

Running title: preoperative breast radiotherapy

**ABSTRACT:**

The purpose of this study was to compare dosimetric parameters of treatment plans among 4 techniques for preoperative single fraction partial breast radiotherapy in order to select an optimal treatment technique. The techniques evaluated were non-coplanar 3D conformal radiation therapy (3D-CRT), non-coplanar intensity modulated radiation therapy (IMRT_NC_), coplanar IMRT (IMRT_CO_) and volumetric modulated arc therapy (VMAT).

The planning CT scans of 16 patients in the prone position were used in this study with the single-fraction prescription doses of 15 Gy for the first 8 patients and 18 Gy for the remaining 8 patients. 6 MV photon beams were designed to avoid the heart and contralateral breast. Optimization for IMRT and VMAT was performed to reduce the dose to the skin and normal breast. All plans were normalized such that 100% of the prescribed dose covered greater than 95% of the clinical target volume (CTV) consisting of gross tumor volume (GTV) plus 1.5 cm margin.

Mean homogeneity index (HI) was the lowest (1.05 ± 0.02) for 3D-CRT and the highest (1.11 ± 0.04) for VMAT. Mean conformity index (CI) was the lowest (1.42 ± 0.32) for IMRT_NC_ and the highest (1.60 ± 0.32) for VMAT. Mean of the maximum point dose to skin was the lowest (73.7 ± 11.5%) for IMRT_NC_ and the highest (86.5 ± 6.68%) for 3D-CRT. IMRT_CO_ showed very similar HI, CI, and maximum skin dose to IMRT_NC_ (differences < 1%). The estimated mean treatment delivery time excluding the time spent for patient positioning and imaging was 7.0 ± 1.0, 8.3 ± 1.1, 9.7 ± 1.0 and 11.0 ± 1.5 min for VMAT, IMRT_CO_ , IMRT_NC_ and 3D-CRT, respectively.

In comparison of all 4 techniques for preoperative single fraction partial breast radiotherapy, we can conclude that non-coplanar or coplanar IMRT were optimal in this study as IMRT plans provided homogeneous and conformal target coverage, skin sparing and relatively short treatment delivery time.

PACS numbers: 81.40.Wx; 87.55.D-;

Keywords: single fraction radiotherapy; partial breast radiotherapy; treatment planning;

1. INTRODUCTION

Postoperative accelerated partial breast irradiation (APBI) using an external beam technique has been commonly used to treat low-risk early breast cancer patients with a smaller volume and larger fractional dose than standard whole breast irradiation (WBI). However, outcome data raise concern that soft tissue fibrosis and cosmetic outcomes may be suboptimal with the external beam technique, possibly related to the large volume of tissue treated to high doses in the postoperative setting. In contrast, a single large dose using intra-operative radiation therapy (IORT) technique has shown encouraging results in toxicity and cosmesis outcomes. While IORT application is limited due to the costly equipment and specialized procedures, a radiosurgery technique using external beam is easily performed by most radiation facilities. Feasibility of single dose partial breast irradiation using a radiosurgery technique has been investigated and shown promising results. Treatment of the intact tumor preoperatively allows a significant reduction in high-dose treatment volume and direct access to more accurately defined high risk target area than postoperative seroma . It also provides an opportunity to assess the response of breast tumors to radiation. For such reasons, a phase I clinical study to evaluate preoperative single fraction partial breast radiotherapy was proposed for early stage breast cancer patients and approved by the institutional review board at Duke University.

Given the relative novelty of this approach, the optimal treatment delivery technique was unknown. Although 3D-CRT has been traditionally used for postoperative APBI, significantly reduced dose to normal surrounding tissue and critical structures with more conformal target coverage have been reported in studies using IMRT or VMAT techniques. However, all prior IMRT or VMAT studies for breast patients did not include constraints for the skin, which, we were concerned, could be a major limitation for single fraction breast radiotherapy.

The purpose of this study was to compare dosimetric parameters of treatment plans in order to select an optimal treatment technique for preoperative single fraction partial breast radiotherapy. The techniques we evaluated in this study were non-coplanar 3D conformal radiation therapy (3D-CRT), non-coplanar intensity modulated radiation therapy (IMRT_NC_), coplanar IMRT (IMRT_CO_) and volumetric modulated arc therapy (VMAT).

1. METHODS AND MATERIALS
2. Simulation

This study included 16 patients who enrolled on a preoperative dose-escalation single fraction partial breast radiotherapy clinical protocol at Duke. Prior to CT scan, all patients had one titanium biopsy marker implanted to identify the tumor. Patients underwent CT (GE Light-speed RT, GE Medical System, Milwakee, WI) scanning with 2.5 mm slice thickness in the prone position on a CDR prone breast board (CDR systems Inc, Calgary, Alberta, Canada), which was placed on top of the CT table and treatment couch. The head was turned to the ipsilateral side and both arms were raised up to hold the handles above the head. The ipsilateral breast (ILB) was allowed to fall naturally through the opening of the prone breast board while the contralateral breast was pulled away with the support of the bridge plate on the board. CT origin was marked on the patient’s skin and the board. Patients also underwent MRI in the prone position (1.5 Tesla GE Signa scanner, GE Medical System) using the open breast array MRI coil (GE Medical system). T1 weighted and dynamic contrast enhanced MR images were acquired to delineate the tumor.

1. Structure segmentations

CT and MR images were imported into the Eclipse treatment planning system (TPS) (Varian Medical systems, Inc., Palo Alto, CA) and registered to align the biopsy marker and soft tissue around the tumor area using manual rigid-body registration. All structures were contoured on the CT. Gross tumor volume (GTV) was identified as the area of enhancement on contrast-enhanced MRI with the assistance of a radiologist specializing in breast imaging . Additional images (mammogram and ultrasound) were also viewed for accuracy and the biopsy marker was confirmed to be in the region of the tumor. A 1.5cm-margin and an additional 0.3cm-margin from the GTV were added to define the clinical target volume (CTV) and the planning target volume (PTV). Both CTV and PTV were modified to keep a minimum 5-mm distance from the skin surface to ensure skin sparing following RTOG protocol 0413 and other single fraction breast radiotherapy studies. It is important to note that the CTV margins for this application were more comprehensive than other single fraction conformal applications Furthermore, a single fraction delivery technique using on-board imaging and target localization based on biopsy marker contributed to minimize patient motion. Therefore, a priority was given to skin sparing over additional margin when generating the PTV. A 3-mm layer along the ILB skin surface was defined as skin following a previous study of breast single-fraction radiotherapy . Heart, lungs, ribs and bilateral normal breasts were segmented on the CT.

1. Treatment planning and optimization

All plans used the anisotropic analytical algorithm (AAA version 10.0.28) for dose calculation with 2.5 mm calculation grid and heterogeneity correction. We intended to set the beam isocenter at the geometrical center of GTV. However, for some patients, the isocenter was set a few centimeters (3 to 8cm) medial to this point in order to avoid collision between the gantry and the patient body/ treatment couch. All plans had gantry, collimator and couch angles set to avoid CB and heart based on beams eye view (BEV) in TPS. No beams entered from the back or side of a patient through a lung. All beams entered directly through the ipsilateral breast to reach to the target effectively. In general, the gantry angles were set to be spread as much as possible (15 degree to 40 degree) among beams within the limited range. If a target was located at the periphery, more beams were arranged to enter from the target side and 1 to 2 beams were arranged to enter from the other sides. Truebeam (Varian Medical Systems) 6 MV photon beams with 600 MU/min dose rate were used for treatment plan dose calculation. All plans were normalized such that 100 % of the prescribed dose covered greater than 95% of the CTV (i.e. V_100%_ > 95%). The single-fraction prescription dose was 15 Gy for the first eight patients and 18 Gy for the remaining patients (first two dose cohorts on the clinical trial).

- *3D-CRT*: Non-coplanar 3D-CRT included 4 to 5 beams following typical APBI 3D-CRT planning, among which 1-3 beams included wedges. The couch rotation was applied to 1-3 beams with a limited angle up to +/- 20 degree to avoid collision. Multi-leaf collimators (MLC) were set to have a 2 mm margin around the PTV or tighter if critical organs such as skin, CB or heart were abutting to the PTV based on BEV. Beam weights and wedge angles were adjusted to provide homogeneous dose distribution and conformal dose to the CTV and PTV.
- *Non-coplanar IMRT (IMRT_NC_)*: Non- coplanar IMRT used dynamic MLC with the same beams utilized for 3D-CRT. Optimization was performed to minimize dose to skin and ILB. Optimization constraints and priorities from Table 1 were initially applied, and they were adjusted as optimization progressed to improve target coverage and to reduce skin dose. Dose-volume constraints for heart, CB and ipsilateral lung (ILL) were set with relatively low priority. No constraint was set for ribs. Optimization process was stopped after about 50 – 70 iterations as the optimization process did not show improved dose-volume histograms (DVH).
- *Coplanar IMRT (IMRT_CO_)*: Coplanar IMRT included 7 beams with couch angle at zero, also using dynamic MLC. The same optimization constraints and process from IMRT_NC_ planning were used.
- *VMAT*: VMAT is a volumetric arc therapy, which utilizes IMRT technique with different MUs delivered at varying dose rate while MLC leafs continuously moving as the gantry is simultaneously rotating at varying speed. RapidArc^TM^ (Varian Medical systems) in Eclipse was used for VMAT planning in this study. Two duplicate sets of partial arcs were included – each set divided into two or three partial arcs to avoid any angle passing through heart and CB. One set rotated counter clockwise with collimator rotation of 30 degree and the other set rotated clockwise with collimator rotation of 330 degree. The same optimization constraints and process from IMRT_NC_ and IMRT_CO_ planning were used.

1. Data analysis

For CTV and PTV, V_95%_, dose homogeneity index (HI) and conformity index (CI) were compared. The ratio of a maximum point dose (D_max_) within the target and the prescribed dose (PD), D_max_/PD, defined the HI. The ratio of prescribed isodose volume (PIV) to the target volume , PIV/TV, defined the CI. The skin D_max_ and dose to 10 cm^3^ (D_10 cm_^3^) of skin as well as V_20%_, V_50%_, V_100%_ and D_1%_ of ILB were compared. For heart, CB and ILL, D_10cm_^3^ was compared. The dose fall off was calculated as the ratio of V_50%_ of ILB to V_100%_ of ILB, V_50%_/V_100%_.

Treatment delivery time was estimated by adding beam-on time and time in between beams. The beam-on time was calculated as the total MUs divided by the dose rate. Even though VMAT utilized a varying dose rate, the maximum dose rate was used for calculation in this study because each arc beam was irradiating the small target with small arc angle and large MUs. To include beam preparation and therapists’ time-out procedure, 20 sec was added for each beam. For a beam with a wedge or couch rotation, 1 min was added to include the time for a therapist to go in and out of the treatment room. For a beam with a wedge and couch rotation, 1 min and 20 sec was added to apply both. Time spent for patient positioning and image guidance was not included in estimating the treatment delivery time.

Comparison of the different techniques was statistically analyzed using the two-tailed paired t-test (p value). Data were considered significant if p was < 0.05.

1. RESULTS

The average volumes of GTV, CTV and PTV were 1.3 ± 0.8 cm^3^ (range 0.2 – 3.5 cm^3^), 46.0 ± 9.3 cm^3^ (range 33.9 – 65.9 cm^3^) and 68.5 ± 12.9 cm^3^ (range 52.3 – 96.5 cm^3^), respectively. The average volume of ILB was 1905.2 ± 829.6 cm^3^ (range 775.9 – 3688.8 cm^3^). The average volume ratio of PTV to ILB was 4.1 ± 1.5 % (range 1.5 – 7.1%).

For target coverage, all plans had V_95%_ of CTV higher than 99.5% and V_95%_ of PTV higher than 95%. 3D-CRT had the highest V_95%_ of CTV and PTV. VMAT had higher CIs and HIs of CTV and PTV than other three techniques. For skin sparing, IMRT_NC_ had the lowest D_10cm_^3^, D_1cm_^3^ and D_max_. Skin D_1cm_^3^ and D_max_ was the highest for 3D-CRT, and skin D_10cm_^3^ was the highest for VMAT. Regarding ILB, VMAT had the lowest V_20%_ and V_50%_, but the highest V_100%_ and D_1%_. IMRT_NC_ and IMRT_CO_ showed similar target conformity, dose homogeneity and normal tissue sparing. 3D-CRT plans yielded higher doses to skin and ILB , but had better target coverage and dose homogeneity than other plans. Heart, CB and ILL received very small doses from all plans in this study. VMAT had the smallest D_10cm_^3^ for heart, CB and ILL compared to the other techniques. The dose fall off was sharper for VMAT than other techniques. The estimated treatment delivery time was about 4 minutes faster for VMAT than 3D-CRT. IMRT_CO_ and IMRT_NC_ were estimated to require 1.3 minutes and 2.7 minutes more than VMAT, respectively.

In the isodose comparison as shown in Figure 1 for one patient, it is notable that the 105% isodose line (magenta) appears only in VMAT as anticipated based on the HI comparison from Table 2. VMAT also shows a smaller 10% isodose line (yellow) than other plans as anticipated given the findings from ILB’s dose comparison (e.g. V_20%_) presented in Table2. DVHs for this patient are also compared in Figure 1 (E) and (F).

1. DISCUSSION

The feasibility of single fraction partial breast radiotherapy has been previously demonstrated . Palta *et. al*  used an IMRT technique and treatment planning MR with a prescription dose of 15 Gy preoperatively whereas the other three studies used 3D-CRT with a prescription dose of 21 Gy postoperatively based on CT images. Our study investigated further the novel preoperative approach in an effort to reduce the volume of normal tissue treatment with the goal of producing acceptable acute toxicity and cosmetic outcomes . Indeed, our study included PTV with the average volume and the maximum volume 30% and 60-70% smaller, respectively, than those from the postoperative single fraction radiotherapy studies . In addition, our study showed V_50%_ and V_100%_ of ILB about 15% and 5%, respectively, whereas postoperative prone APBI studies showed about a V50 of approximately 40-50% and V100 of ~20-30%. Similarly, postoperative supine APBI studies, which reported acceptable cosmetic outcome, showed a V50 of ~35-40% and V100 of ~10-15% . Such substantial improvement in V_50%_ and V_100%_ of ILB were attributed to the preoperative approach of this study. The studies performed by Bondiau *et al*. proposed preoperative breast stereotactic body radiotherapy (SBRT) and postoperative conventional breast radiotherapy . Their studies did not report ILB volumes, but showed similar mean PTV volume. Therefore, we presume a similar benefit to preoperative radiotherapy from their studies as well.

Dose to the skin could be a significant limiting factor for this single-fraction approach compared to other APBI planning studies. Timmerman suggested 14.4Gy to < 10 cc of skin and 16Gy to skin D_max_ as dose constraints for single fraction treatment to avoid skin ulceration . Our study showed D_10cm_^3^ of skin getting less than 50% of the prescription dose (7.5 – 9 Gy), but D_max_ higher than 16Gy with all four plans for one patient and with 3D-CRT for another patient. Bondiau et a*l.* reported grade 3 dermatologic dose limiting skin toxicity for one patient with the prescription of 17.5 Gy for a 3-fraction SBRT. They associated this toxicity not with the dese level but with the tumor size and the sagging patch of skin. Iaccarino *et al.* reported no skin toxicity with an average of 15.2 Gy to 3% of the skin volume , and our study had less than 15.2 Gy to 3% of the skin volume for all patients with all plans. Pinnaro *et al.* stopped their study prematurely due to unsatisfactory cosmesis rate or grade 2 skin toxicity such as erythema, fibrosis and necrosis, and noted a mean skin dose of ~ 5.4Gy . The mean skin dose in our study was less than 3 Gy for all patients with all plans. Hoppe *et al.* reported acute Grade 2 or higher skin toxicity at skin D_max_ of 24-30 Gy in 3-4 fractions, which is equivalent to 13-18 Gy in a single fraction for skin using an a/b of 2.3 . Our study showed skin D_max_ > 13Gy for 15 patients in 3D-CRT, 5 patients in IMRT_NC_ and VMAT, and 4 patients in IMRT_CO_, but all less than 17 Gy. The mean skin D_max_ was about 1 Gy greater in the 2^nd^ dose cohort (18 Gy) than the 1^st^ dose cohort (15 Gy). Treatment planning may be more challenging for the 3^rd^ dose cohort – 21 Gy. Though this section evaluated our plans compared to other similar studies reporting skin toxicity, it should be noted that the studies from Hoppe *et al.* and Timmermen were conducted in different clinical scenarios. Though our data compares favorably with other studies, more conservative constraints may be required for this application. Ultimately, we will need to await outcomes from our recently completed phase I study to make this determination. In addition, it should be noted that there could be some uncertainty in the skin dose analysis due to the differences in skin definition. Timmerman, Pinnaro *et al.* and Hoppe *et al.* did not specify how skin was defined in their manuscripts whereas Iaccarino *et al.* used 3 mm and Bondiau et a*l.* used 5 mm thickness to define the skin. Dose calculation accuracy could be another uncertainty in evaluating skin dose as it is in the build-up region. However, AAA is known to provide accurate dose calculation for the build-up region .

Regarding rib fracture, Wilkinson *et al* reported 2% of patients getting rib fractures with the maximum rib dose ranging from 28 to 33.6 Gy delivered over 4 fractions in breast brachytherapy. It is equivalent to 15.3 to18 Gy in a single fraction for rib using an a/b of 3. Our study showed the maximum rib dose of higher than 15.3, but all less than 16.5 Gy for 2 patients. Dunlap *et al*. suggested less than 30 ml to receive 30 Gy in 3 – 5 fraction to avoid rib fracture based on lung SBRT. Again, using an a/b of 3, it is equivalent to 15 to 18 Gy in a single fraction. Our study had no patient receiving more than 15 Gy for 0.1 cm^3^ of rib. We anticipate that rib fracture were not a significant risk for patients enrolled in this study. However, we will have to await clinical outcomes from our phase I study for more definitive answers.

Without long-term follow up, it is hard to predict toxicities associated with normal structures such as heart, lung, and CB. This was incorporated into consideration in the designs of the phase I clinical trial and treatment plans. The prescription dose started at 15 Gy for the 1^st^ cohort and increased to 18 Gy in order to follow acute toxicities, and further increased to 21 Gy for the last cohort. Treatment beams in plans were designed to avoid such normal structures and optimization constraints were also used to limit the dose. Consequently, only small volumes of normal structures received very low dose.

3D-CRT and IMRT_NC_ required at least 4 or 5 beams to achieve acceptable target coverage. Adding more beams (total 6 beams or more) increased the estimated treatment delivery time without noticeable improvement in dosimetric results. VMAT and IMRT_CO_ have an advantage over 3D-CRT and IMRT_NC_ in that therapists do not need to go in and out of the room in between beams. IMRT_CO_ was initially tested with various numbers of beams. IMRT_CO_ with 7 beams provided comparable results to IMRT_NC_ and estimated treatment delivery time fell in between VMAT and IMRT_NC_. VMAT with one set of partial arcs was initially intended for this study, but the target coverage was noticeably inferior (e.g. CI > 2). VMAT achieved comparable dosimetric results by utilizing duplicate sets of partial arcs. VMAT with triplicate sets of partial arcs showed insignificant improvement in dosimetric parameters compared to VMAT with duplicate sets while the estimated treatment delivery time increased by 50%. It should be noted that the time spent for patient setup and imaging prior to treatment delivery could be much longer. However, the time increase from IGRT part should be uniform across techniques. Once images are approved by an attending physician, it is critical to deliver the treatment quickly to achieve accurate delivery without patient motion.

Prior APBI plan comparison studies showed that IMRT spared heart, CB and lungs more than 3D-CRT and VMAT, and VMAT spared better than 3D-CRT . Nevertheless, we included 3D-CRT in this study for comparison with other previous single fraction breast radiotherapy studies . In this study, heart, CB and lungs received very small dose in all plans. Yet, VMAT had smaller doses to heart, CB, ribs and ILL than IMRT or 3D-CRT due to the reduced scatter with smaller total MUs. Regarding ILB, Shaitelman *et al*. noted that VMAT plans reduced dose to ILB at the expense of increased dose elsewhere . Our study showed the same trend that VMAT had sharp dose fall off resulting in reduced dose to ILB, but target dose homogeneities and skin doses were higher compared to IMRT and 3D-CRT.

For the actual treatment of the 16 patients, IMRT_NC_ or IMRT_CO_ was used. Contrasting to the dosimetric study reported here, beams were allowed to exit through a small portion of the heart and/or CB for some patients. Consequently, the mean D_10cm_^3^ of heart and CB were 5% and 2% greater in the actual plans than in IMRT plans from this study. Doses to other structures in the actual plans were comparable to IMRT plans from this study.

1. CONCLUSION

The treatment planning technique was not specifically delineated in our phase I preoperative single fraction partial breast radiotherapy protocol. Therefore, we compared four different techniques to determine the optimal treatment planning technique. All plans achieved acceptable target coverage. 3D-CRT yielded significantly higher skin dose than the other plans. VMAT offered the shortest estimated treatment delivery time and better sparing of normal tissue except skin, but yielded less dose homogeneity within target and conformity in this study. Based on the results of our current study, there was no treatment planning technique that provided better dosimetric parameters for all structures with a shortest treatment delivery time. However, we can conclude that either non-coplanar IMRT or coplanar IMRT are the optimal treatment technique for preoperative single fraction partial breast radiotherapy as the IMRT plans provided homogeneous and conformal target coverage, and skin sparing with relatively short treatment delivery time.

**References**

1. Baglan K L, Sharpe M B, Jaffray D, Frazier R C, Fayad J, Kestin L L, et al. Accelerated partial breast irradiation using 3D conformal radiation therapy (3D-CRT). Int J Radiat Oncol Biol Phys. 2003; 55(2): 302-311.

2. Formenti S. External-Beam Partial-Breast Irdiation. Semin Radiat Oncol. 2005; 15: 92-99.

3. Ragsi J, Ben-David M, Moran J, Marsh R, Griffith K, Hayman J, et al. Adverse Cosmesis in a Protocol Investigating IMRT with Active Breathing Control for Accelerated Partial Breast Irradiation (APBI). Int J Radiat Oncol Biol Phys. 2008; 72(1): S153.

4. Whelan T, Olivotto I, Parpia S, Berrang T, Kim D, Kong I, et al. presented at the ASTRO 54th Annual Meeting. Boston, MA, 2012 (unpublished).

5. Jagsi R, Ben-David M A, Moran J M, Marsh R B, Griffith K A, Hayman J A, et al. Unacceptable Cosmesis in a Protocol Investigating Intensity-Modulated Radiotherapy With Active Breathing Control for Accelerated Partial-Breast Irradiation. Int J Radiat Oncol Biol Phys. 2010; 76(1): 71-78.

6. Hepel J T, Tokita M, MacAusland S G, Evans S B, Hiatt J, Price L, et al. Toxicity of 3D-CRT for Accelereated Partial Breast Irradiation. Int J Radiat Oncol Biol Phys. 2009; 75(5): 1290-1296.

7. Veronesi U, Orecchia R, Luini A, Galimberti V, Zurrida S, Intra M, et al. Intraoperative radiotherapy during breast conserving surgery: a study on 1,822 cases treated with electrons. Breast cancer research and treatment. 2010; 124(1): 141-151.

8. Vaidya J S, Joseph D J, Tobias J S, Bulsara M, Wenz F, Saunders C, et al. Targeted intraoperative radiotherapy versus whole breast radiotherapy for breast cancer (TARGIT-A trial): an international, prospective, randomised, non-inferiority phase 3 trial. Lancet. 2010; 376(9735): 91-102.

9. Jozsef G, Luxton G and Formenti S. Application of radiosurgery principles to a target in the breast: A dosimetric study. Med Phys. 2000; 27(5): 1005-1010.

10. Iaccarino G, Pinnaro P, Landoni V, Marzi S, Soriani A, Giordano C, et al. Single fraction partial breast irradiation in prone position. J Exp Clin Cancer Res. 2007; 26(4): 543-552.

11. Pinnaro P, Arcangeli S, Giordano C, Arcangeli G, Impiombato F, Pinzi V, et al. Toxicity and cosmesis outcomes after single fraction partial breast irradiation in early stage breast cancer. Radiation Oncology. 2011; 6(1): 155.

12. Palta M, Yoo S, Adamson J D, Prosnitz L R and Horton J K. Preoperative Single Fraction Partial Breast Radiotherapy for Early-Stage Breast Cancer. Int J Radiat Oncol Biol Phys. 2012; 82(1): 37-42.

13. Nichols E M, Dhople A A, Mohiuddin M, Flannery T, Yu C and Regine W F. Comparative analysis of the post-lumpectomy target volume versus the pre-lumpectomy tumor volume for early-stage breast cancer: implications for the future. Int J Radiat Oncol Biol Phys. 2010; 77(1): 197-202.

14. Horst K, Fero K, Ikeda D, Daniel B and Dirbas F. Defining an optimal role for breast magnetic resonance imaging when evaluating patients otherwise eligible for accelerated partial breast irradiation. Radiation Oncology. 2013; 108(2): 220-225.

15. Azoury F, Heymann S, Acevedo C, Spielmann M, Vielh P, Garbay J-R, et al. Phase II trial of 3D-conformal accelerated partial breast irradiation: Lessons learned from patients and physicians’ evaluation. Radiother Oncol. 2012; 103(2): 193-198.

16. Scabderbeg D, Yashar C, White G, Rice R and Pawlicki T. Evaluation of three APBI techniques under NSABP B-39 guidelines. J App Clin Med Phy. 2010; 11(1): 274-280.

17. Qiu J-J, Chang Z, Wu Q J, Yoo S, Horton J and Yin F-F. Impact of Volumetric Modulated Arc Therapy Technique on Treatment With Partial Breast Irradiation. Int J Radiat Oncol Biol Phys. 2010; 78(1): 288-296.

18. Khan A J, Kirk M C, Mehta P S, Seif N S, Griem K L, Bernard D A, et al. A dosimetric comparison of three-dimensional conformal, intensity-modulated radiation therapy, and MammoSite partial-breast irradiation. Brachytherapy. 2006; 5(3): 183-188.

19. Moon S H, Shin K H, Kim T H, Yoon M, Park S, Lee D-H, et al. Dosimetric comparison of four different external beam partial breast irradiation techniques: Three-dimensional conformal radiotherapy, intensity-modulated radiotherapy, helical tomotherapy, and proton beam therapy. Radiother Oncol. 2009; 90(1): 66-73.

20. Rusthoven K E, Carter D L, Howell K, Kercher J M, Henkenberns P, Hunter K L, et al. Accelerated Partial-Breast Intensity-Modulated Radiotherapy Results in Improved Dose Distribution When Compared With Three-Dimensional Treatment-Planning Techniques. Int J Radiat Oncol Biol Phys. 2008; 70(1): 296-302.

21. Shaitelman S F, Kim L H, Yan D, Martinez A A, Vicini F A and Grills I S. Continuous Arc Rotation of the Couch Therapy for the Delivery of Accelerated Partial Breast Irradiation: A Treatment Planning Analysis. Int J Radiat Oncol Biol Phys. 2011; 80(3): 771-778.

22. Bergom C, Prior P, Kainz K, Morrow N, Ahunbay E, Walker A, et al. A phase I/II study piloting accelerated partial breast irradiation using CT-guided intensity modulated radiation therapy in the prone position. Radiation Oncology. 2013; 108(2): 215-219.

23. Andrews D, Scott C, Sperduto P, Flanders A, Gaspar L, Schell M, et al. Whole brain radiation therapy with or without stereotactic radiosurgery boost for patients with one to three brain metastases: phase III results of the RTOG 9508 randomised trial. Lancet. 2004; 363(9422): 1665-1672.

24. Yoo S, O'Daniel J, Horton J, Blitzblau R and Yin F. A Dual-Isocenter Technique for Image Guidance of Prone-Breast Radiation Therapy. Int J Radiat Oncol Biol Phys. 2011; 81(2, Supplement): S824-S825.

25. A randomized phase III study of conventional whole breast irradiation (WBI) versus partial breast irradiation (PBI) for women with stage 0, I, or II breast cancer. (RTOG protocol 0413, 2009).

26. Bondiau P-Y, Bahadoran P, Lallement M, Birtwisle-Peyrottes I, Chapellier C, Chamorey E, et al. Robotic Stereotactic Radioablation Concomitant With Neo-Adjuvant Chemotherapy for Breast Tumors. International Journal of Radiation Oncology*Biology*Physics. 2009; 75(4): 1041-1047.

27. Otto K. Volumetric modulated arc therapy: IMRT in a single gantry arc. Med Phys. 2008; 35(1): 310-317.

28. Shaw E, Scott C, Souhami L, Dinapoli R, Kline R, Loeffler J, et al. Single dose radiosurgical treatment of recurrent previously irradiated primary brain tumors and brain metastases: final report of RTOG protocol 90-05. Int J Radiat Oncol Biol Phys. 2000; 47(2): 291-298.

29. Formenti S C, Hsu H, Fenton-Kerimian M, Roses D, Guth A, Jozsef G, et al. Prone Accelerated Partial Breast Irradiation After Breast-Conserving Surgery: Five-year Results of 100 Patients. Int J Radiat Oncol Biol Phys. 2012(0).

30. Wen B, Hsu H, Formenti-Ujlaki G F, Lymberis S, Magnolfi C, Zhao X, et al. Prone Accelerated Partial Breast Irradiation After Breast-Conserving Surgery: Compliance to the Dosimetry Requirements of RTOG-0413. Int J Radiat Oncol Biol Phys. 2012; 84(4): 910-916.

31. Formenti S C, Truong M T, Goldberg J D, Mukhi V, Rosenstein B, Roses D, et al. Prone accelerated partial breast irradiation after breast-conserving surgery: Preliminary clinical results and dose–volume histogram analysis. Int J Radiat Oncol Biol Phys. 2004; 60(2): 493-504.

32. Leonard C E, Carter D L, Kercher J M, Howell K, Henkenberns P, Tallhamer M, et al. Prospective trial of accelerated partial breast intensity-modulated radiotherapy. Int J Radiat Oncol Biol Phys. 2007; 67(5): 1291-1298.

33. Bondiau P-Y, Courdi A, Bahadoran P, Chamorey E, Queille-Roussel C, Lallement M, et al. Phase 1 Clinical Trial of Stereotactic Body Radiation Therapy Concomitant With Neoadjuvant Chemotherapy for Breast Cancer. International Journal of Radiation Oncology*Biology*Physics. 2013; 85(5): 1193-1199.

34. Timmerman R D. An overview of hypofractionation and introduction to this issue of seminars in radiation oncology. Semin Radiat Oncol. 2008; 18(4): 215-222.

35. Hoppe B S, Laser B, Kowalski A V, Fontenla S C, Pena-Greenberg E, Yorke E D, et al. Acute Skin Toxicity Following Stereotactic Body Radiation Therapy for Stage I Non–Small-Cell Lung Cancer: Who's at Risk? Int J Radiat Oncol Biol Phys. 2008; 72(5): 1283-1286.

36. Chakarova R, Gustafsson M, Bäck A, Drugge N, Palm Å, Lindberg A, et al. Superficial dose distribution in breast for tangential radiation treatment, Monte Carlo evaluation of Eclipse algorithms in case of phantom and patient geometries. Radiother Oncol. 2011; (Article in press)(1-6).

37. Wilkinson J B, Martinez A A, Chen P Y, Ghilezan M I, Wallace M F, Grills I S, et al. Four-year results using balloon-based brachytherapy to deliver accelerated partial breast irradiation with a 2-day dose fractionation schedule. Brachytherapy. 2012; 11(2): 97-104.

38. Coroller T, Mark R, Lewis J, Baldini E, Chen A, Colson Y, et al. Low incidence of chest wall pain with a risk-adapted lung stereotactic body radiation therapy approach using three or five fractions based on chest wall dosimetry. PLoS One. 2014; 9(4): e94859.

39. Dunlap N E, Cai J, Biedermann G B, Yang W, Benedict S H, Sheng K, et al. Chest Wall Volume Receiving &gt;30 Gy Predicts Risk of Severe Pain and/or Rib Fracture After Lung Stereotactic Body Radiotherapy. International Journal of Radiation Oncology*Biology*Physics. 2010; 76(3): 796-801.

**Figure and figure legend**


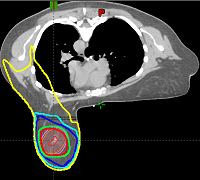

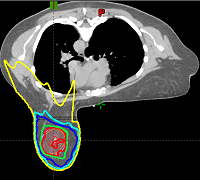

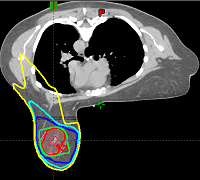

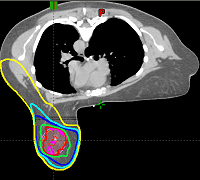

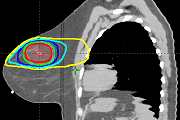

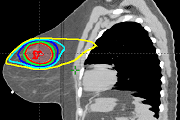

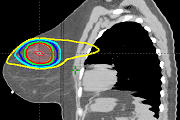

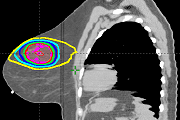


**(A-1)**

**(A-2)**

**(B-1)**

**(B-2)**

**(C-1)**

**(C-2)**

**(D-1)**

**(D-2)**


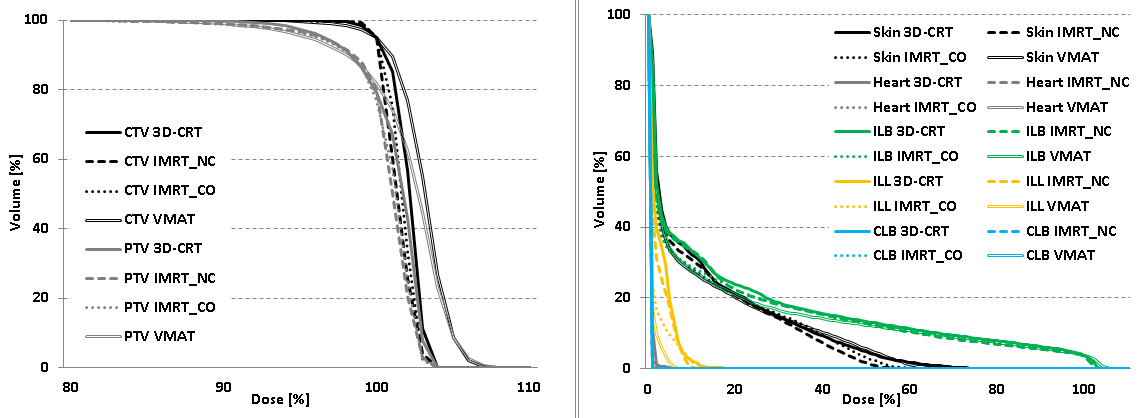


**(E)**

**(F)**

Figure 1. Isodose distributions of 3D-CRT (A-1 & A-2), IMRT_NC_ (B-1 & B-2), IMRT_CO_ (C-1 & C-2) and VMAT (D-1 & D-2) in axial view (top row) and sagittal view (bottom row) and dose-volume histograms of CTV and PTV (E) and normal tissues (F). Line colors indicate – magenta 105%, red 100%, green 90%, blue 50%, cyan 30% and yellow 10% isodose lines. GTV is contoured in red, CTV in orange and PTV in pink.

**Tables and captions**

Table 1. Initial dose-volume constraints and priorities for IMRT and VMAT optimization

| Structure | Dose-volume constraints | Relative priority |
| --- | --- | --- |
| PTV | D_100_ ≥ 95%; D_max_ ≤ 105% | 80-120 |
| CTV | D_100_ ≥ 98%; D_max_ ≤ 102% | 120-130 |
| Skin | D_max_ ≤ 70%;  V_50%_ ≤ 2% | 150  80 |
| ILB | V_100%_ ≤ 2-7%; V_50%_ ≤ 10-15%; V_20%_ ≤ 20-30%; | 50 |
| CB | D_max_ ≤ 1%; | 30 |
| Heart | D_max_ ≤ 1%; | 30 |
| ILL | D_max_ ≤ 20-30%; V_10%_ ≤ 2-7% | 30 |
| Normal tissue | Doses between 2mm and 2cm from PTV boundary to fall off from 100% to 50% of prescription | 80 |

Table 2.Mean dosimetric parameters ± standard deviation from 3D-CRT, IMRT_NC_, IMRT_CO_, and VMAT.

|  |  | 3D-CRT | | IMRT_NC_ | IMRT_CO_ | VMAT |
| --- | --- | --- | --- | --- | --- | --- |
| CTV | V_95%_ | 99.8 ± 0.4 | | 99.5 ± 0.6^*^ | 99.5 ± 0.6^*^ | 99.5 ± 0.6^*^ |
|  | HI | 1.05 ± 0.02 | | 1.07 ± 0.04^*^ | 1.07 ± 0.03^*^ | 1.11 ± 0.04^*†‡^ |
|  | CI | 1.56 ± 0.27 | | 1.42 ± 0.32 | 1.44 ± 0.30 | 1.60 ± 0.32 |
| PTV | V_95%_ | 98.5 ± 1.4 | | 96.3 ± 2.0^*^ | 96.5 ± 1.5^*^ | 96.4 ± 1.8^*^ |
|  | HI | 1.05 ± 0.02 | | 1.08 ± 0.05^*^ | 1.07 ± 0.04^*^ | 1.12 ± 0.02^*†‡^ |
|  | CI | 1.04 ± 0.17 | | 0.95 ± 0.20 | 0.96 ± 0.21 | 1.07 ± 0.22^†‡^ |
| Skin | D_10 cm_^3^ (%) | 45.9 ± 7.1 | | 41.9 ± 5. 9^*^ | 43.8 ± 6.5 | 46.3 ± 7.42^†‡^ |
|  | D_1 cm_^3^ (%) | 72.9 ± 9.6 | | 59.2 ± 10.3^*^ | 61.1 ± 11.9^*^ | 64.2 ± 11.7^*†^ |
|  | D_max_ (%) | 86.5 ± 6.7 | | 73.7 ± 11.5^*^ | 74.3 ± 12.1^*^ | 76.3 ± 12.5^*^ |
| ILB | V_20%_ (%) | 28.5 ± 8.5 | | 27.0 ± 7.8^*^ | 26.4 ± 8.3^*^ | 23.5 ± 7.5^*†‡^ |
|  | V_50%_ (%) | 15.3 ± 5.3 | | 14.3 ± 3.9 | 14.8 ± 5.5 | 14.1 ± 5.6^*‡^ |
|  | V_100%_ (%) | 4.3 ± 1.6 | | 3.8 ± 1.3^*^ | 3.9 ± 1.4 | 4.4 ± 1.9^† ‡^ |
|  | D_1%_ (%) | 103.7 ± 1.8 | | 103.9 ± 2.5^*^ | 104.0 ± 2.3^*^ | 105.6 ± 2.8^*^ |
| Heart | D_10cm_^3^(%) | 2.5 ± 1.7 | | 1.8 ± 1.6^*^ | 1.6 ± 1.4^*^ | 1.3 ± 1.1^*†‡^ |
| CB | D_10cm_^3^(%) | 1.0 ± 0.5 | | 0.7 ± 0.3^*^ | 0.8 ± 0.3^*†^ | 0.6 ± 0.2^*‡^ |
| ILL | D_10cm_^3^(%) | 11.4 ± 8.3 | | 13.2 ± 10.8 | 11.8 ± 9.8 | 9.6 ± 9.9^† ‡^ |
| Ribs | D_max_ (%) | 33.6 ± 33.0 | | 34.3 ± 36.6 | 33.7 ± 35.8 | 30.4 ± 36.6^†‡^ |
| Dose fall off | V_50%_/V_100%_ of ILB | 3.7 ± 0.8 | | 3.9 ± 0.8 | 4.0 ± 1.1 | 3.3 ± 0.8^*† ‡^ |
| Delivery time (min) | | 11.0 ± 1.5 | 9.7 ± 1.0^*^ | | 8.3 ± 1.1^*†^ | 7.0 ± 1.0^*†‡^ |
| * p value < 0.05 compared to 3D-CRT where p = the two-tailed paired t-test  † p value < 0.05 compared to IMRT_NC_  ‡ p value < 0.05 compared to IMRT_CO_ | | | | | | |
